# Genomic Characterization and Pathogenicity of a Novel Birnavirus Strain Isolated from Mandarin Fish (*Siniperca chuatsi*)

**DOI:** 10.3390/genes16060629

**Published:** 2025-05-24

**Authors:** Hetong Zhang, Dandan Zhou, Junjian Dong, Yunyun Yan, Shanshan Liu, Xing Ye, Jianguo He, Chengfei Sun

**Affiliations:** 1 Key Laboratory of Tropical and Subtropical Fisheries Resource Application and Cultivation, Ministry of Agriculture and Rural Affairs, Pearl River Fisheries Institute, Chinese Academy of Fishery Sciences, Guangzhou 510310, China; zhanght@prfri.ac.cn (H.Z.); dongjj@prfri.ac.cn (J.D.); 20222170546@pgs.hebau.edu.cn (Y.Y.); yexing@prfri.ac.cn (X.Y.); 2 Key Laboratory of Aquatic Animal Immune Technology of Guangdong Province, Pearl River Fisheries Institute, Chinese Academy of Fishery Sciences, Guangzhou 510310, China; 3 State Key Laboratory of Biocontrol, Southern Marine Sciences and Engineering Guangdong Laboratory (Zhuhai), School of Marine Sciences, Sun Yat-sen University, Guangzhou 510310, China; zhoudd5@mail2.shsu.edu.cn; 4 College of Oceanography, Agriculture University of Hebei, Qinhuangdao 066000, China; 5 School of Ecology, Sun Yat-sen University, Guangzhou 510275, China; liushansh@mail.sysu.edu.cn; 6 School of Life Sciences, Sun Yat-sen University, Guangzhou 510275, China

**Keywords:** birnavirus, mandarin fish, pathogenicity, disinfectant

## Abstract

Background: Birnaviruses infect a wide range of aquatic and terrestrial hosts, including several economically important fish species. This study aimed to isolate and characterize a novel birnavirus strain from mandarin fish (*Siniperca chuatsi*), a high-value freshwater species in Chinese aquaculture. Methods: A novel strain, designated mandarin fish birnavirus (MFBV), was isolated from diseased fish and propagated in SCK cells. The complete genome was determined using high-throughput sequencing and RACE. Viral replication kinetics, tissue distribution, and pathogenicity were assessed through in vitro infection, RT-qPCR, histopathology, and experimental challenges. In addition, disinfectant sensitivity and environmental stability were evaluated. Results: The MFBV genome comprises two segments (A: 3539 bp; B: 2719 bp), and phylogenetic analysis revealed close relatedness to largemouth bass birnavirus (LBBV) and *Lates calcarifer* birnavirus (LCBV). MFBV displayed rapid replication in SCK cells, completing a replication cycle in 8–10 h. In juvenile and fry fish, an experimental infection caused acute disease with cumulative mortality ranging from 41.8% to 83.6%, with fry showing higher susceptibility. Viral RNA was detected in multiple tissues (7.9 × 10^6^–7.9 × 10^7^ copies/μg RNA), and histopathological lesions were observed in the intestine, spleen, and kidney. MFBV was highly sensitive to glutaraldehyde (20 ppm), while other disinfectants showed reduced efficacy. Viral half-life ranged from 36.5 to 144.5 h at room temperature. Conclusions: These findings demonstrate that MFBV can induce acute systemic infection in mandarin fish. The results offer new insights into the genomic and biological features of birnaviruses, contributing to improved disease management and viral taxonomy.

## 1. Introduction

The family *Birnaviridae* includes seven genera. Among these, the *Aquabirnavirus*, *Avibirnavirus* and *Blosnavirus* infect vertebrates, while the remaining genera, *Dronavirus*, *Entomobirnavirus*, *Ronavirus*, and *Telnavirus*, infect insects, rotifers, or mollusks (International Committee on Taxonomy of Viruses, Virus Taxonomy: 2023 Release). Notably, infectious pancreatic necrosis virus (IPNV, genus *Aquabirnavirus*) and infectious bursal disease virus (IBDV, genus *Avibirnavirus*) are extensively studied birnaviruses that primarily affect salmonids and poultry, respectively.

Birnaviruses have bisegmented double-stranded RNA (dsRNA) genomes, totaling about 6 kbp. Segment A, approximately 3.1–3.5 kbp in length, encodes a polyprotein of VP2-(X)-VP4-VP3. Segment B, around 2.7 kbp in length, encodes the viral RNA-dependent RNA polymerase (RdRp, VP1). The polyprotein undergoes autocatalytic cleavage by the VP4 protease, yielding the precursor VP2 (preVP2), VP4, and VP3. The preVP2 is subsequently matured by cleaving the C-terminal small peptides, and then matured VP2 constitutes the capsid, while VP3 is a ribonucleoprotein [1,2].

Birnaviruses are non-enveloped, single-layered capsid dsRNA viruses, distinct from the double-layered capsid of most dsRNA viruses. Their capsid is an icosahedron, composed of 260 outer trimers, arranged with a triangulation number of T = 13, and has a diameter of approximately 60–70 nm, as observed in representative birnaviruses such as IBDV and IPNV [3,4].

Building on metaviromic sequencing data and structural characterization of birnaviruses, recent studies have uncovered additional fish-pathogenic members of this family. Two novel fish birnaviruses, namely largemouth bass birnavirus (LBBV) reported in 2022 and *Lates calcarifer* birnavirus (LCBV), identified in 2019, have been described. LBBV has been associated with high virulence and results in massive mortality in largemouth bass (*Micropterus salmoides*), whereas LCBV infection has only been linked to mild clinical signs in seabass (*L. calcarifer*) [5,6]. These findings suggest that birnaviruses appear to be increasingly prevalent in farmed fish in Southeast Asia in recent years. Given the increasing reports of birnavirus infections in aquaculture species, this study aimed to identify and characterize a novel birnavirus strain isolated from mandarin fish—a commercially important species in Chinese aquaculture, with annual production reaching approximately 480,000 metric tons in 2023 [7].

In this study, we obtained the complete genome of this virus and characterized its replication dynamics and virulence, this virus was named mandarin fish birnavirus (MFBV). Multiple novel birnavirus genomes have been sequenced in recent years [5,6,8], but systematic comparative analyses of their genomic architectures remain scarce, which impedes the taxonomic refinement of emerging birnaviruses. We therefore conducted a detailed analysis of the genome structure of MFBV and other birnaviruses. The inactivation efficiency of the disinfectant on MFBV was also tested to provide suggestions for practical application.

## 2. Materials and Methods

### 2.1. Animals and Cells

Healthy juvenile mandarin fish weighing 10 ± 2 g and fry mandarin fish weighing 0.02 g were obtained from Foshan, Guangdong Province, China. The *Siniperca chuatsi* kidney (SCK) cells were isolated and maintained in our laboratory [9]. Cells used in this study were at approximately passage 100. Routine maintenance was performed using high-glucose DMEM supplemented with 10% fetal bovine serum.

### 2.2. Isolation of the Viral Strain

In the original case, in July 2022, at a fish farm in Nansha District, Guangzhou, mandarin fish (weighing 15–20 g) were observed floating on the water and dying within a few hours. A cumulative mortality rate reached 80% after three weeks. The naturally infected fish exhibited inappetence, lethargy, and reduced swimming activity. Post-mortem observations revealed yellowish mucus in the intestines. Subsequent screenings for infectious spleen and kidney necrosis virus (ISKNV), mandarin fish ranavirus (MRV), *Siniperca chuatsi* rhabdovirus (SCRV), and nervous necrosis virus (NNV) by a commercial service provider were all negative. Bacterial detection was performed by a commercial service provider Yingyu Co. by using species-specific PCR assays to exclude infections caused by *Aeromonas hydrophila*, *Aeromonas schubertii*, *Aeromonas veronii*, *Edwardsiella tarda*, and *Flavobacterium columnaris*. Spleen and kidney tissues from affected fish were sampled. Ten times the volume of PBS (Gibco, Waltham, MA, USA) was added, and the tissues were thoroughly homogenized. The homogenate was filtered through a 0.45 μm filter (Millipore, Burlington, MA, USA) to remove bacterial contamination, and then inoculated into SCK cells at a ratio of 1:100 (*v*/*v*) and incubated at 27 °C. Cytopathic effect (CPE)-positive cells were identified and images were captured using an Observer.Z1 inverted microscope (ZEISS, Baden-Württemberg, Germany) at indicated time points. Infected cells underwent three freeze–thaw cycles and were vortexed for 10 s to homogenize. The yielded virus was regarded as the first-passage virus. Subsequent rounds of infection in SCK cells generated viral progeny, sequentially referred to as the second passage, third passage, and so forth. The viral culture medium was harvested as the virus stock and stored at −80 °C.

### 2.3. Library Construction, Sequencing, and Genome Assembly

RNA was extracted from MFBV-infected SCK cells, and the RNA library was constructed by using the RNA library prep kit for Illumina (New England Biolabs, Ipswich, MA, USA) according to the manufacturer’s protocol. A total of 500 ng of high-quality total RNA was used for library construction. rRNA depletion was conducted prior to library preparation, poly(A) enrichment was not performed. The library was sequenced on the Illumina NovaSeq platform (Illumina, San Diego, CA, USA) at a commercial sequencing institution (Majorbio, Shanghai, China), generating 150 bp paired-end reads. The raw sequencing reads were filtered and quality-trimmed using fastp (version 0.21.0) to remove low-quality bases and adapters. After quality filtering and trimming, a total of 52,721,968 clean reads were obtained and used for downstream analysis. The resulting high-quality reads were de novo assembled using SPAdes (v3.15.4) in metagenomic mode (‘-meta’) with default settings. Contigs longer than 1000 bp were annotated against the NCBI nonredundant protein (nr) database using DIAMOND (blastx, version 2.0.11) with a stringent E value of 10^−5^ to eliminate false-positive results.

### 2.4. Amplification of Genome Terminal Sequence

Viral RNA was extracted from MFBV-infected cell cultures using a Monarch Total RNA Miniprep Kit (New England Biolabs, MA, USA). Poly(A) tailing of viral genome RNA using *E. coli* Poly(A) polymerase (New England Biolabs, MA, USA). The SMARTer RACE kit (Takara, Kyoto, Japan) was employed to amplify the terminal sequences of the genome. First-strand cDNA synthesis was performed using a modified oligo(dT) primer with an adapter sequence at the 5′ end. The 5′ and 3′ ends of the cDNA were then amplified using nested specific primers and the universal primer mix provided in the kit. RACE PCR reactions were conducted in a 40 μL volume using Q5 High-Fidelity 2× Master Mix (20 μL, New England Biolabs, MA, USA), UPM primer (4 μL), gene-specific primer (2 μL), template RNA (2 μL), and nuclease-free water (12 μL). The thermocycling conditions were: 98 °C initial denaturation for 30 s, followed by 35 cycles of 98 °C for 10 s, 65 °C for 20 s, and 68 °C for 30 s. The amplified products were analyzed by gel electrophoresis, and bands of interest were purified and sequenced using Sanger sequencing technology (Shenggong, Shanghai, China). The obtained sequences were then assembled and analyzed to determine the terminal sequences of the RNA virus genome. The specific primers used are listed below:

SegmentA-3′-outerCCCTCACCCAGAGGAGCACCAAACTSegmentA-3′-innerCAAGCCCCTGCACCACCAGAGTTTGSegmentA-5′-outerCGGAGAGTACCCTGCTGACCAGTCTSegmentA-5′-innerAGGCCTTCTTCAGGTCCTGTGAGGTSegmentB-3′-outerAAAGAAGCAGAAGCAGCAGCCGACCSegmentB-3′-innerACCGACGACTGGGGAGAAGCATCAGSegmentB-5′-outerGCTCGGGCTTGTGCATGGGGTAGTASegmentB-5′-innerCGACGAGTACAGCCAGACTGGGGAG

The complete genome sequence of MFBV has been deposited in GenBank under the accession numbers PP786692.1 (Segment A) and PP786693.1 (Segment B).

### 2.5. Analysis of the MFBV Genome

Multiple sequence alignment was performed using Kalign [10]. The birnavirus sequences used in the phylogenetic analysis were the following: largemouth bass birnavirus (LBBV), MW727622.1 and MW727622.1; *Lates calcarifer* birnavirus (LCBV), MK103419.1 and MK103420.1; blotched snakehead virus (BSNV), AJ459382.1 and AJ459383.1; infectious pancreatic necrosis virus (IPNV), AJ622822.1 and AJ622823.1; infectious bursal disease virus (IBDV), MZ888508.1 and MZ888507.1; Drosophila melanogaster birnavirus (DBV), GQ342962.1 and GQ342963.1; rotifer birnavirus (RBV), FM995220.1 and FM995221.1; Tellina virus 1 (TV-1), AJ920335.1 and AJ920336.1; and Drosophila x virus (DXV), U60650.1 and NC_004169.1. The best-fit substitution model for phylogenetic analysis was determined using the “Find Best DNA/Protein Models” tool implemented in MEGA software (Version 11). Among the evaluated models, the LG+γ distributed with invariant sites (LG+G+I) model was selected as the most appropriate for the dataset, based on the lowest Bayesian Information Criterion (BIC) score. Phylogenetic trees were constructed using the maximum likelihood (ML) method with the following parameters: substitution model, LG+G+I; ML heuristic method, Nearest Neighbor Interchange (NNI); and branch support, 1000 bootstrap replicates.

### 2.6. Transmission Electron Microscopy

SCK cells were inoculated with MFBV at an MOI of 5. At 16 h post-infection (hpi), cells were gently scraped, centrifuged at 500× *g* for 3 min, and then fixed in 2.5% glutaraldehyde at room temperature for 30 min and then transferred to 4 °C for overnight storage (Solarbio, Beijing, China). Following osmium tetroxide (Ted Pella, Redding, CA, USA) post-fixation for 1 h at 4 °C, uranyl acetate (Ted Pella, USA) was applied to enhance membrane contrast. Epoxy resin (SPI, TX, USA) served as the embedding medium. Resin polymerization was conducted in two stages: 45 °C for 24 h followed by 60 °C for 24 h. The ultrathin sections were visualized using a transmission electron microscope (Hitachi HT7800, Tokyo, Japan). Imaging was performed using a transmission electron microscope at an accelerating voltage of 80 kV. Fields were scanned at 3000× *g* magnification to locate virus particles, and images were captured at 20,000× *g* magnification.

Spleens and kidneys were sampled from moribund fish following intraperitoneal infection with MFBV. These fish exhibited typical clinical signs including intestinal mucus accumulation, ascites, and cutaneous congestion, especially in the head region. The spleen and kidney tissues were cut into 1 mm^3^ pieces and fixed in 2.5% glutaraldehyde. Secondary fixation was performed with osmium tetroxide. The fixed specimens were dehydrated by incubation in a series of ethanol (Sinopharm chemical reagent, Shanghai, China) solutions (30%, 50%, 70%, 80%, 95%, and 100%) followed by 100% acetone (Sinopharm chemical reagent, China). After resin infiltration and embedding, the samples were sectioned to 80 nm thickness (Leica, Wetzlar, Germany), mounted on copper mesh, and post-stained with lead citrate (Ted Pella, CA, USA). The ultrathin sections were visualized using a Hitachi HT7800 transmission electron microscope.

### 2.7. Virus Titer Assays Measurement of Viral Titer

To assess viral infectivity, SCK cells were plated in 48-well plates containing 200 μL of medium per well. Third-passage MFBV samples were harvested at defined time points, subjected to three cycles of freezing and thawing, and briefly vortexed to ensure sample uniformity. Serial 10-fold dilutions were prepared, and each dilution was inoculated into six replicate wells. Cells was monitored up to 7 days post-inoculation, with wells deemed positive upon characteristic cytopathic changes. 50% tissue culture infective dose (TCID_50_) values were determined using the Spearman–Kärber method via the TCID₅₀ calculator (Marco Binder, University of Heidelberg) [11], and expressed as mean ± standard error from three independent experiments.

### 2.8. Challenge Experiment

All animal experiments were approved by the Laboratory Animal Ethics Committee of Pearl River Fisheries Research Institute (Approval No. LAEC-PRFRI-2023-05). Fish were maintained under a 14 h light/10 h dark photoperiod. Juvenile mandarin fish were fed with live bait (*Cirrhinus molitorella*) at 2× body count per day, with the baitfish length 30–50% of the mandarin fish. Fry were fed newly hatched *C. molitorella* fry (approximately 50% of the fry’s body length) at a feeding density of 10–20× body count per day to ensure ad libitum availability. Juveniles were acclimated in the laboratory for 1 week, and fry were acclimated for 2 days, prior to the challenge. All fish exhibited normal swimming and feeding behavior. Before the challenge, viscera from three juvenile and fry fish were sampled and examined for the absence of ISKNV, MRV, SCRV, and MFBV by PCR. All fish used in challenge and sampling experiments were randomly assigned to groups. In the juvenile mandarin fish intraperitoneal injection challenge study, we used unbalanced control group experimental designs: independent experiment 1 included 15 fish in the experimental group and 10 fish in the control group; independent experiment 2 included 19 fish in the experimental group and 10 fish in the control group; and independent experiment 3 included 20 fish in the experimental group and 10 fish in the control group. Fish were kept in 150 L tanks at temperatures ranging from 28 to 30 °C. Each fish received an intraperitoneal injection of 10^9^ TCID_50_ 3rd-passage MFBV suspended in 0.2 mL of cell culture medium. Additionally, ten fish were injected with the same volume of untreated cell cultures to serve as controls. Fish were considered moribund if they exhibited loss of swimming behavior and equilibrium, failed to respond to tactile stimuli, and showed markedly reduced operculum movement. These signs typically persisted for tens of minutes prior to death. Fish meeting these criteria were euthanized by immersion in ice water for a minimum of 10 min. Death was confirmed by the absence of operculum movement and visible skin pallor. Fish mortality was monitored daily for three weeks, with cumulative mortality data compiled from the three separate experiments.

In the juvenile and fry mandarin fish immersion challenge study, fish were immersed in water containing a virus concentration of 10^7^ TCID_50_/mL for 20 min, then transferred to 150 L tanks at 28 to 30 °C. Control group fish were immersed in an equal volume of serum-containing medium under identical conditions. In the juvenile fish immersion challenge study, we included 16 fish in the experimental group and 10 fish in the control group in each independent experiment. In the fry mandarin fish immersion challenge study, independent experiment 1 included 70 fish in the experimental group and 55 fish in the control group; independent experiment 2 included 123 fish in the experimental group and 98 fish in the control group; and independent experiment 3 included 92 fish in the experimental group and 80 fish in the control group. For fry fish, the study concluded at 3 dpi.

Kaplan–Meier survival analysis was used to compare mortality rates between the experimental and control groups. Detailed artificial infection data and statistical analyses are provided in Supplementary Table S1.

### 2.9. Absolute Quantitative RT-PCR

The fish in the treatment group were exposed to the same infection dose and method as used in the intraperitoneal challenge experiment. From this group, 9 surviving or moribund fish were randomly selected for tissue sampling, and 14 tissues, along with intestinal content samples, were collected at 16 hpi, including spleen, pronephros, mesonephros, metanephros, pyloric caeca, intestine, intestinal contents, stomach, thymus, heart, skin, gill, liver, gonad, and brain. Tissue samples were preserved in 1.5 mL of RNAlater solution, and RNA from the samples was isolated using a Monarch Total RNA Miniprep Kit (New England Biolabs, MA, USA). RNA was incubated at 65 °C for 5 min and promptly placed on ice to denature RNA double strands and secondary structures. Reverse transcription was performed using Induro Reverse Transcriptase (New England Biolabs, USA) according to the manufacturer’s protocol.

Absolute quantitative PCR with primers for MFBV segment A was performed (Applied Biosystems 7300 Real-Time PCR System, Waltham, CA, USA) to quantify the total copies of viral genome and mRNA. For standard curve construction, the segment A PCR amplification product, containing the T7 promoter sequence, was used for in vitro transcription with HiScribe T7 (New England Biolabs, USA) according to the manufacturer’s instructions. The resulting RNA was quantified using a BioTek Cytation instrument (Agilent, Santa Clara, CA, USA). A 10-fold serial dilution series (ranging from 10⁻^3^ to 10⁻^8^) was prepared as standards using nuclease-free water (Thermo Fisher Scientific, Waltham, MA, USA) as the diluent. Each dilution was aliquoted and stored at −80 °C to prevent degradation. The equation of the standard curve is y = −3.4445x + 44.159. RT-qPCR was performed in a total reaction volume of 20 μL containing 0.2 μM primers, 1 μL of cDNA, 10 μL of 2 × SYBR Green Premix (Takara, Kyoto, Japan), and 7.2 μL of RNase-free water. The following settings were used: 40 cycles of amplification for 10 s at 95 °C and 30 s at 65 °C. Each sample was run in technical triplicates. Primers used were MFBVsegA-qPCR-F: CTGGATAGCCAGGAACGACC and MFBVsegA-qPCR-R: GTTGTCGGCGTACACTTCCT

### 2.10. Histopathological Sectioning and H&E Staining

Intestine, spleen, and metanephros tissues from dying individuals were fixed with formalin and acetic acid fixative overnight and preserved with 70% ethanol. Tissues were dehydrated using a graded ethanol series to remove water. The dehydration steps were as follows: 70% ethanol for 1 h, 80% ethanol for 1 h, 95% ethanol for 1 h, and 100% anhydrous ethanol (two changes, 1 h each). The dehydrated tissues were cleared in xylene (two changes, 1 h each). The cleared tissues were then infiltrated with molten paraffin wax at 60 °C for 1 h and allowed to solidify at room temperature. Paraffin-embedded tissues were sectioned at a thickness of 5 μm. The slides were dried at 60 °C for 2 h. The sections were stained using the H&E protocol as follows: we immersed the slides in Harris hematoxylin solution (BASO, Zhuhai, China) for 5 min, rinsed in running tap water for 2 min, differentiated in 1% acid alcohol (1% HCl in 70% ethanol) for 30 s, and rinsed in running tap water for 2 min. The slides were then immersed in 0.5% eosin Y solution (Sigma-Aldrich, Burlington, MA, USA) for 5 s, followed by dehydration in 95% ethanol and 100% anhydrous ethanol for 2 min. Clear sections in xylene (two changes, 2 min each). The slices were then observed under the microscope.

### 2.11. Determine the Inactivation Efficiency of Disinfectants

All disinfectants were diluted to the appropriate concentrations using sterile ultrapure water and added to the virus suspension (11 Log^10^ TCID_50_/mL). The mixture was immediately vortexed for 10 s for homogenization and centrifuged at 100× *g* for 3 s to collect the liquid from the tube lid. After incubation at 25 °C for 30 min, the viral titer was immediately assayed using the method described above. The cells were incubated with the virus-infected cell culture supernatants without disinfectant treatment as positive controls, while the same concentration of disinfectant was used as a control to exclude any effect on cell activity. A detailed description of the disinfectant concentrations and reaction volumes is provided in Supplementary Table S1.

### 2.12. Determination of the Stability of MFBV Suspension

MFBV-infected SCK cell cultures, harvested at 24 h post-infection with a titer of 11.73 Log^10^ TCID_50_/mL, were incubated at room temperature under dark conditions for up to 90 days to assess viral stability and infectivity over time. At predetermined time points, the samples were briefly vortexed for 3 s to homogenize any precipitated material. Aliquots were then immediately collected and titrated in SCK cells using the TCID_50_ assay, as described in Section 2.7.

## 3. Results

### 3.1. Tissue Electron Microscopy

**Figure 1:**
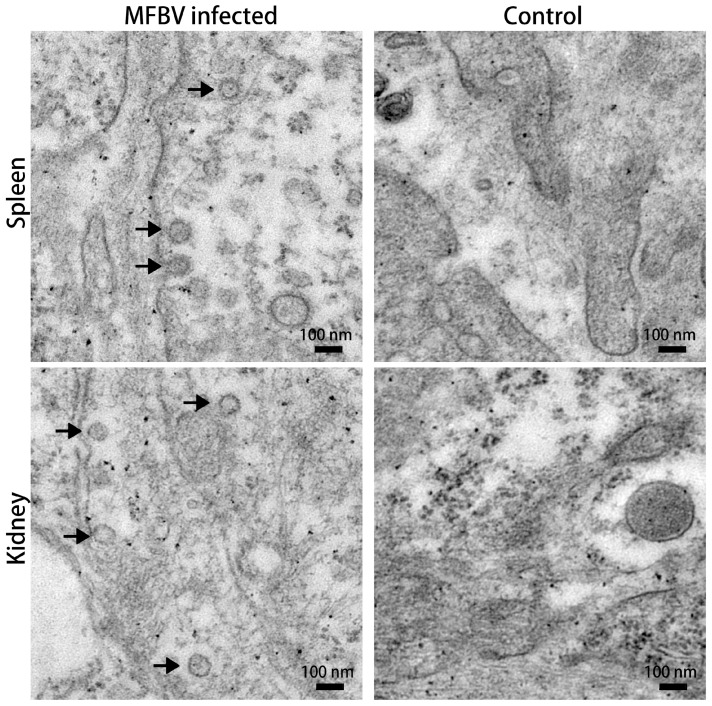
TEM micrographs of spleen and kidney tissue from the infected fish and control fish. Arrows indicate virus-like particles with a polyhedral morphology. The magnifications are ×20,000.

Spleen and kidney samples from the infected fish were collected for transmission electron microscopy (TEM). Virus-like particles with diameters of approximately 60–80 nm were observed in both tissues, with some displaying a polyhedral morphology (Figure 1).

### 3.2. Assessment of Cytopathic Effect

**Figure 2:**
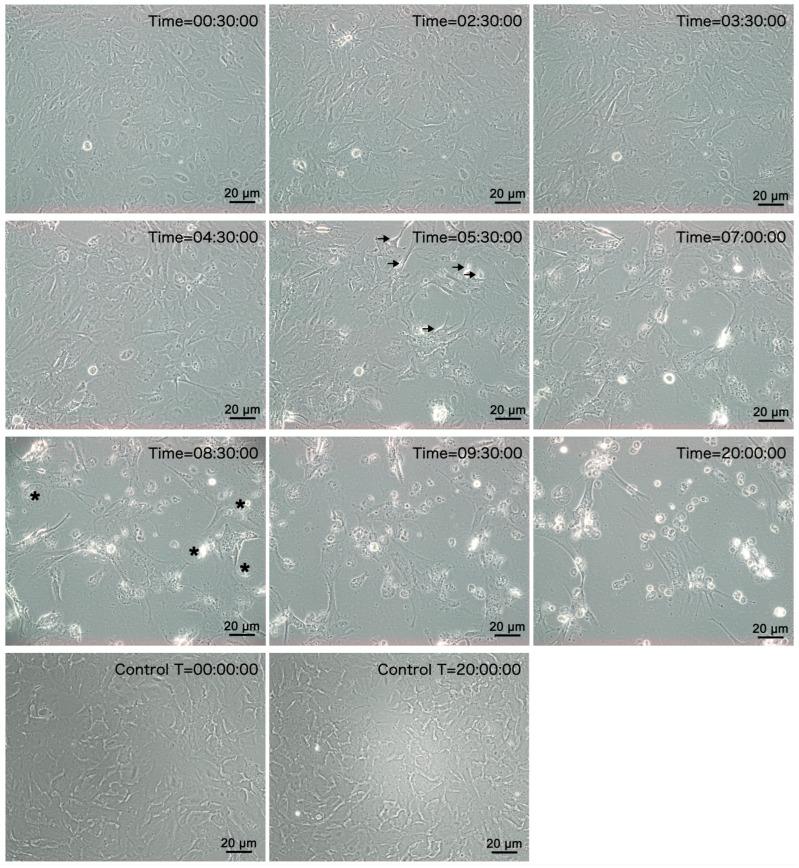
Phase contrast microscopic images depicting CPE in SCK cells elicited by the 3rd passage of MFBV at different hpi. The arrows indicate morphological and refractive index changes in infected cells at 5.5 hpi. The asterisks indicate cell rounding and detachment from the substrate at 8.5 hpi. Scale bar represents 20 μm.

Upon inoculation with tissue homogeates, highly refractive regions appeared at the edges of cells at 5.5 hpi, indicating morphological and/or refractive index changes in infected cells (Figure 2). By 8.5 hpi, obvious CPE manifested, characterized by cell rounding and detachment from the substrate. At 8.5 hpi, approximately 75% of the cells exhibited cytopathic effects (CPE), including cell rounding and detachment. By 20 hpi, CPE was observed in over 90% of the cell monolayer. The number of cells showing CPE peaked at 20 hpi (Figure 2). Subsequent passages of the viral culture fluid to fresh SCK cell monolayers showed a similar pattern of CPE. In contrast, the control SCK cell monolayer remained normal and intact throughout the incubation period (Figure 2).

### 3.3. TEM of Infected Cells

**Figure 3:**
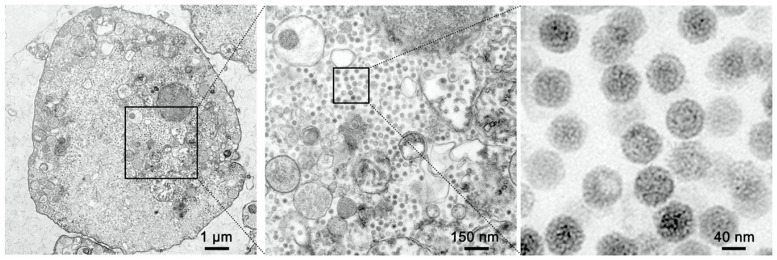
TEM micrographs of MFBV-infected SCK cells. The images on the right side are magnified views from the boxed region in the adjacent left images. The magnifications are ×3000, ×20,000, ×40,000 in the order of the images.

Furthermore, we verified viral replication and characterized virion morphology in the cytoplasm of MFBV-infected SCK cells through transmission electron microscopy. The diameter of virus particles, measured from different symmetry axes, ranged from 65 nm to 80 nm. These particles exhibited an icosahedral capsid with an electron-dense core. Upon examination at higher magnification, the particles displayed a rough surface structure (Figure 3).

### 3.4. The Organization of MFBV Genome

**Figure 4:**
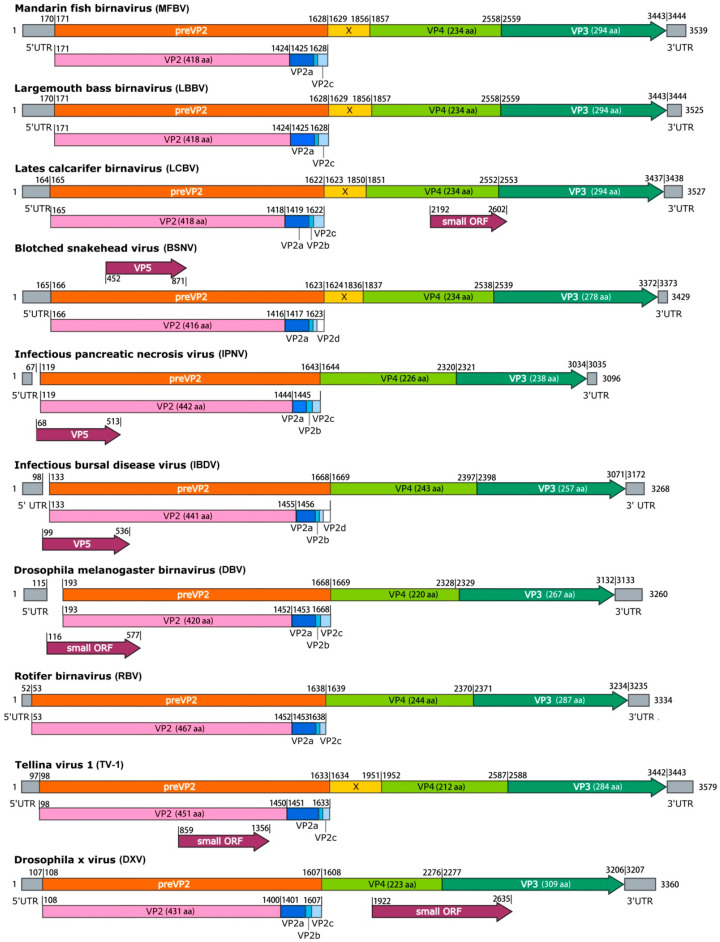
Schematic representation of the segment A genome organization of MFBV and other representative birnaviruses. The additional pink graphics illustrating processing of preVP2. Polyprotein cleavage sites are indicated by vertical bars and identified by nucleotide positions. Numbers at the 3′ ends indicate the full length of segment A.

The complete genome of MFBV was obtained through high-throughput sequencing and RACE. Segment A of MFBV is 3539 bp in length (GC content: 57.33%), and segment B is 2719 bp (GC content: 56.34%). Open reading frames (ORFs) were identified based on homology alignment with reference birnavirus sequences. Segment A contains a single ORF spanning nucleotides 171–3443, encoding a polyprotein (preVP2-X-VP4-VP3). MFBV segment A harbors an X gene located between preVP2 and VP4. The polyprotein precursor (preVP2) is predicted to generate three small peptides (43 aa, 7 aa, and 18 aa) during maturation. No overlapping small ORF (analogous to VP5 in other birnaviruses) was identified in the MFBV genome. While segment B encodes VP1 (nucleotides 97–2667) (Figure 4). The isolated virus strain was designated as MFBV-22TIE (accession: PP786692.1; PP786693.1).

**Figure 5:**
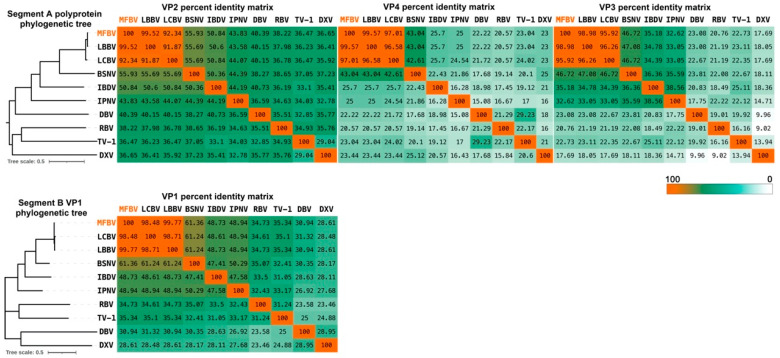
Distance tree representing the phylogenetic relationships of the polyprotein and VP1 within the *Birnaviridae* family. The percent identity matrices are generated based on VP2, VP3, VP4, and VP1 amino acid sequences.

Phylogenetic analysis of polyprotein and VP1 was conducted on the amino acid level, incorporating representatives from seven genera in the *Birnaviridae* family. Additionally, percent identity matrices were generated for the four proteins to examine their sequence identity. The results showed that both the polyproteins and VP1 of MFBV, LBBV, and LCBV clustered into a single evolutionary branch, with the four mature proteins displaying sequence homologies from 95.92% to 99.57%. Furthermore, regardless of the protein analyzed, MFBV appears to be phylogenetically distant from other birnaviruses. Among other branches, the genetically closest virus was BSNV, another fish birnavirus, with VP2, VP4, VP3, and VP1 showing sequence homologies of only 55.93%, 43.03%, 46.72%, and 61.36%, respectively. IPNV exhibited even lower homologies, ranging from only 25% to 48.94% (Figure 5). Among these four proteins, VP2 and VP1 are relatively conserved due to their requirement for conserved folding and topological structure [12]. Conversely, VP4 and VP3 have diverged significantly during birnavirus evolution, exemplified by the mere 20% sequence homology between MFBV and DXV (Figure 5).

### 3.5. Virus Titer Assays

**Figure 6:**
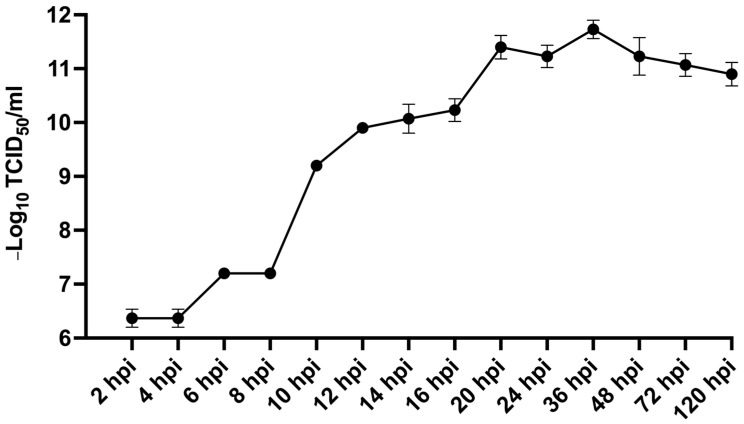
Infectious dynamics of MFBV in SCK cells. Viral titers of the 3rd passage of MFBV at indicated time points were measured and expressed as TCID_50_/ml. Each titer value represents the mean ± SE derived from three biological replicates. Partial SE is hidden within the data points.

To investigate the infectious dynamics of MFBV, we harvested virus cell culture at various time points post-infection. We then inoculated this viral fluid into SCK cells to obtain viral titers. The results revealed a rapid increase in viral titers from 8 to 10 hpi, rising from 1.58 × 10^7^ to 1.58 × 10^9^ TCID_50_/mL. By 20 hpi, the titers had reached 2.51 × 10^11^ TCID_50_/ml and remained stable near peak levels up to 48 hpi. These findings suggest that a single cycle of replication for MFBV takes approximately 8–10 h (Figure 6).

### 3.6. MFBV Artificial Challenge

**Figure 7:**
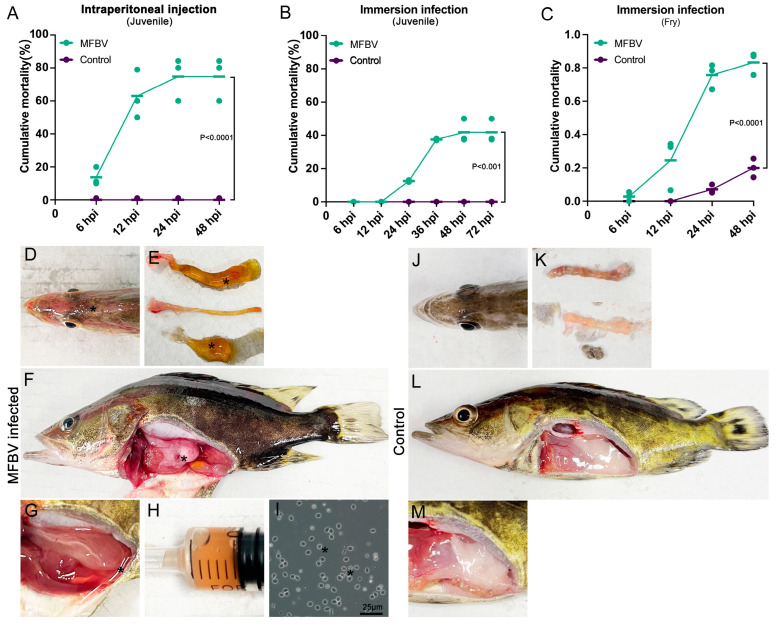
(**A**–**C**) The cumulative mortality rates in MFBV-infected mandarin fish; mean ± SD from three independent experiments, Kaplan–Meier survival analysis was used to compare mortality rates between the experimental and control groups. (**A**) Juvenile mandarin fish as the experimental subjects, challenged via intraperitoneal injection. (**B**) Juvenile mandarin fish as the experimental subjects, challenged via immersion. (**C**) Fry mandarin fish as the experimental subjects, challenged via immersion. (**D**–**I**) Symptoms of MFBV-infected mandarin fish. (**D**) Congestion of the head skin (denoted by asterisks). (**E**) From top to bottom, the intestine filled with mucus, the intestine after removing the mucus, and the mucus itself. Asterisks indicate the yellow mucus. (**F**) Anatomical view of MFBV-infected mandarin fish. Asterisks indicate visceral congestion. (**G**–**H**) Asterisks indicate pale red ascites. All ascites (~0.5 mL) account for approximately 5% of body weight. (**I**) Asterisks indicate red blood cells observed in the ascitic fluid smear. (**J**) Healthy fish skin. (**K**) Healthy fish intestine and contents. (**L**) Anatomical view of healthy mandarin fish. (**M**) The abdominal cavity of healthy fish.

The virulence of the third-passage MFBV from SCK cell was evaluated in juvenile (~10 g of body weight) and fry (~0.02 g of body weight) mandarin fish. Juvenile mandarin fish were subjected to intraperitoneal injection and immersion challenges. In the intraperitoneal injection challenge study, mortality induced by MFBV began at 6 hpi, rapidly escalating to 62.9% by 12 hpi, and reaching 74.7% at 24 hpi, then persisting throughout the subsequent 14-day observation period. No mortality was observed in the intraperitoneal injection control groups across three independent experiments, and the *p*-values were <0.0001 between the infected group and the control group (Kaplan–Meier survival analysis) (Figure 7A). In the juvenile mandarin fish immersion challenge, mortality was initially observed between 12 and 24 hpi, reaching 37.5% at 36 hpi, and further increasing to 41.8% at 48 hpi, ceasing throughout the observation period. No mortality was observed among the control fish, and the *p*-values were <0.001 between the infected group and the control group (Kaplan–Meier survival analysis) (Figure 7B). Both challenge methods led to similar clinical manifestations in all infected fish, including congestion of the head skin, visceral congestion, ascites containing red blood cells comprising 2–5% of body weight, and intestines containing a jelly-like mucus (Figure 7D–I). Fish in the control group remained healthy (Figure 7J–M). In the bath challenge of fry fish, mortality rapidly increased during 6–24 hpi, reaching 83.6% by 48 hpi. However, at 48 hpi, the control group also exhibited modest mortality of 19.9%. Consequently, the mortality rate in MFBV-infected fry was adjusted using Abbott’s formula, yielding a corrected value of 79.5%. The *p*-values between the infected group and the control group were <0.0001 (Kaplan–Meier survival analysis) (Figure 7C). In the immersion experiment with the same titer, the virulence of MFBV to fry was higher than that of juvenile fish. Furthermore, the viscera homogenates of deceased fish tested positive for MFBV by RT-qPCR, while those of control fish tested negative. When inoculated with the homogenates from cases, SCK cell cultures developed characteristic cytopathic effects. These results collectively demonstrate the pathogenicity of MFBV in both juvenile and fry mandarin fish.

### 3.7. Tissue Distribution of MFBV

**Figure 8:**
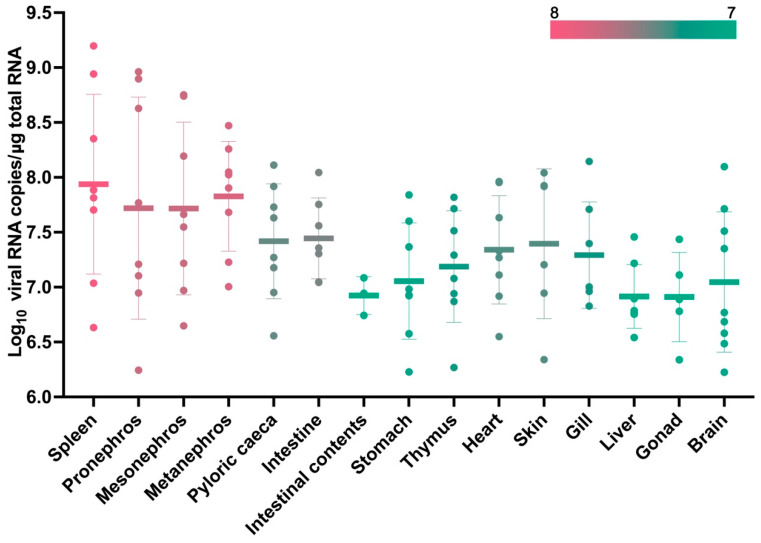
RT-qPCR measured the copy number of MFBV segment A RNA in 14 tissues and intestinal content of MFBV-infected mandarin fish. The color scale indicates the level of viral copy numbers. Mean ± SD from tissues of nine fish.

We further measured the total copy number of MFBV genomic and messenger RNA from infected juvenile fish using RT-qPCR. The highest viral RNA copy number was observed in the spleen tissue, with an average of 8.71 × 10^7^ viral RNA copies per μg of total RNA. Additionally, the three kidney organs, including pronephros, mesonephros, and metanephros, also exhibited high levels of viral RNA, ranging from 5.25 × 10^7^ to 6.76 × 10^7^ viral RNA copies per μg of total RNA. In the digestive systems, the intestine and pyloric caeca showed approximately 2.75 × 10^7^ viral RNA copies per μg of total RNA. For skin, heart, gill and thymus tissue, the copy numbers reached 2.51 × 10^7^, 2.19 × 10^7^, 1.95 × 10^7^, and 1.55 × 10^7^ viral RNA copies per μg of total RNA, respectively. Other tissues, such as the stomach, liver, gonad, brain, and intestinal contents, showed lower concentrations of approximately 7.94 × 10^6^ viral RNA copies per μg of total RNA (Figure 8). These results indicate that during intraperitoneal infection, the highest copy numbers were detected in the spleen and kidneys. Nevertheless, viral RNA was also detected in a range of other organs, highlighting the systemic nature of infection.

### 3.8. Histopathology

**Figure 9:**
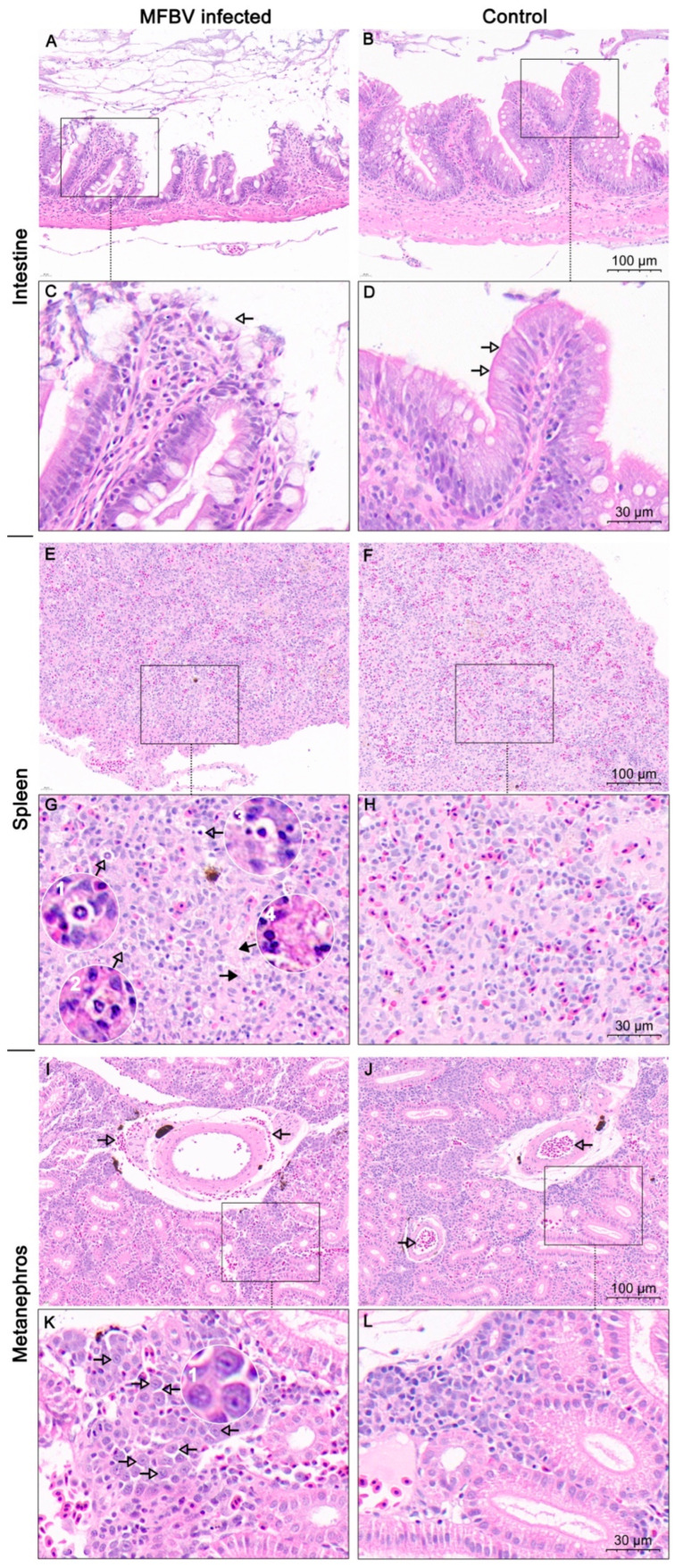
H&E staining analysis of MFBV-infected or healthy mandarin fish tissue sections. (**A**,**C**) Intestinal tissue of MFBV-infected mandarin fish, where arrows indicate damage to the simple columnar epithelium layer and rupture of goblet cells. (**B**,**D**) Intestinal tissue of control mandarin fish, displaying a complete mucosal layer, simple columnar epithelial cell layer, and visible goblet cell vacuoles. (**E**,**G**) Spleen tissue of MFBV-infected mandarin fish. The hollow arrow and magnified view (G1–G3) indicate nuclear condensation (pyknosis), while the solid arrow and image (G4) show nuclear loss and vague cellular outlines (karyolysis and cell death) and the areas that lose structure detail. (**F**,**H**) Control mandarin fish spleen tissue. (**I**,**K**) Arrows in (**I**) indicate red blood cells surrounding the artery. Arrows in (**K**) and magnified view (K1) indicate abnormal chromatin condensation. (**J**,**L**) Control kidney tissue, where blood cells are contained within the arteries (arrow) and nuclei morphology appears normal.

In intestine tissue sections infected with MFBV, evident damage to villus structures was observed, characterized by shedding of the simple columnar epithelium layer and rupture of goblet cells, leading to exposure of the lamina propria to the intestinal lumen (Figure 9A,C). In contrast, healthy intestinal villi displayed a dense layer of simple columnar epithelial cells on the surface, along with clearly distinguishable goblet cell vacuoles (Figure 9B,D). Tissue section observation also revealed varying degrees of chromatin condensation in some spleen cells, along with instances of nuclear loss and vague cellular outlines (Figure 9E, G1–G4). In addition, several areas had a loss of structure detail (Figure 9G). The abnormal cell morphology, indicative of pyknosis and karyolysis, was widely recognized in MFBV-infected spleen tissue, indicating cell death, but not observed in control tissues (Figure 9E–H). Furthermore, in MFBV-infected kidney tissue, red blood cell infiltration was observed around the arteries, whereas in control sections, blood cells, including red blood cells, were contained within the arteries. However, no pathological changes in the arteries were observed, and the source of the leaked red blood cells could not be conclusively determined from the slices (Figure 9I–L). Additionally, a group of cells with chromatin condensation and larger intercellular spaces aggregated into clusters and displayed multifocal distribution in MFBV-infected kidney tissue. Conversely, these features were not observed in the control kidney tissue (Figure 9I–L). These pieces of evidence suggest that MFBV infection results in damage to multiple visceral organs.

### 3.9. Efficacy of Disinfectants at Inactivating MFBV

**Figure 10:**
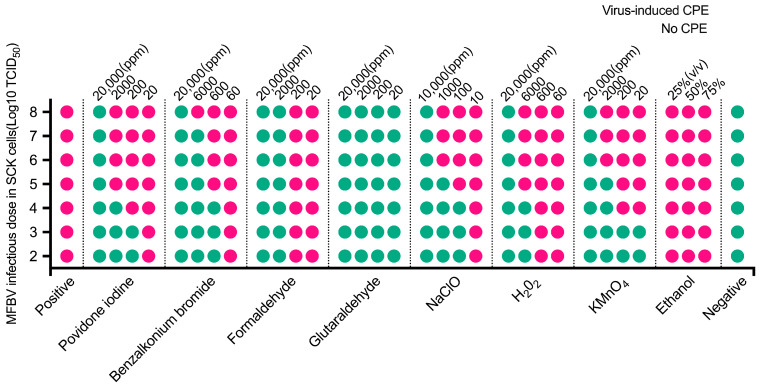
Titer determination of disinfectants treated MFBV virus suspension within SCK cells. Virus-induced CPE positivity is indicated by pink dots, while negativity is indicated by green dots. The data were obtained from three biological replicates, and the results were consistent across experiments.

MFBV virus suspension was treated with various disinfectants at different final concentrations, followed by viral titer determination in SCK cells. The results showed that aldehyde-based disinfectants have strong inactivation efficacy against MFBV, with glutaraldehyde being the most effective. A concentration of 20 ppm glutaraldehyde reduced the titer by 8 Log^10^ TCID_50_, whereas formaldehyde achieved the same reduction need 2000 ppm final concentrations (Figure 10). Povidone-iodine, an iodine-based disinfectant, reduced the titer by 4 Log^10^ TCID_50_ at 2000 ppm, and 20,000 ppm resulted in an 8 Log^10^ TCID_50_ reduction. For benzalkonium bromide, 600 ppm reduced the titer by 4 Log^10^ TCID_50_, 6000 ppm by 7 Log^10^ TCID_50_, and 60,000 ppm by 8 Log^10^ TCID_50_. Oxidizing agents such as sodium hypochlorite reduced the titer by 4 Log^10^ TCID_50_ at 100 ppm, 5 Log^10^ TCID_50_ at 1000 ppm, and 8 Log^10^ TCID_50_ at 10000 ppm. Hydrogen peroxide reduced the titer by 4 Log^10^ TCID_50_ at 6000 ppm and 8 Log^10^ TCID_50_ at 20,000 ppm, while potassium permanganate achieved a reduction of 5 Log^10^ TCID_50_ at 2000 ppm and 8 Log^10^ TCID_50_ at 20,000 ppm. Overall, glutaraldehyde and benzalkonium bromide achieved disinfection efficacy of over 3 log-unit need concentrations at the 10^2^ ppm range. While povidone-iodine, formaldehyde, sodium hypochlorite, hydrogen peroxide, and potassium permanganate required concentrations in the 10^3^ ppm range to achieve similar efficacy. Notably, ethanol showed no inactivation effect on MFBV at concentrations of 25%, 50%, and 75%.

### 3.10. Stability of MFBV

**Figure 11:**
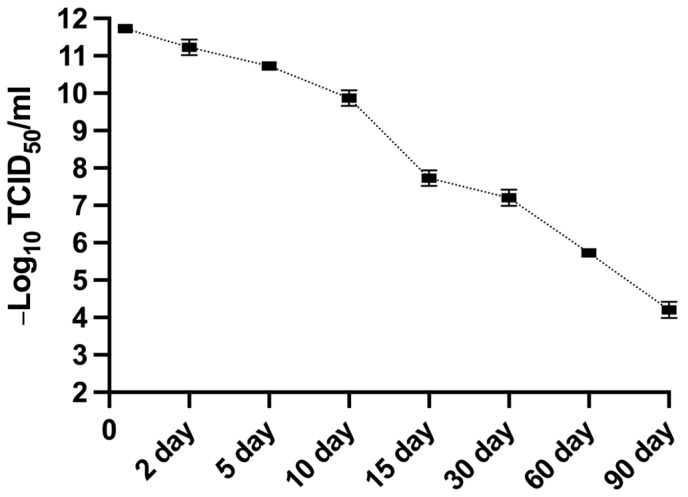
The MFBV suspension was incubated for 90 days, and viral titers were measured at various time points to evaluate the stability of MFBV. Viral titers are expressed as TCID_50_/ml. Each titer value represents the mean ± SE derived from three biological replicates. Partial SE is hidden within the data points.

MFBV-infected SCK cell suspension was collected at 2 dpi and incubated for up to another 90 days to monitor the titer decay. The half-life of MFBV appears to show biphasic decay of short half-life and long half-life at different stages. To analyze changes in the infectious half-life of the virus over time, we adjust our testing intervals. The initial viral titer was 11.73 Log^10^ TCID_50_/ml, and by the end of the experiment on day 90 at room temperature, the titer had decreased to 4.2 Log^10^ TCID_50_/ml (Figure 11). Using data from days 0, 5, 10, and 15 as segment 1, we calculated that it took approximately 15.2 days for the titer to decrease by 3 log units, corresponding to a 99.9% reduction in infectious viral particles. The half-life of the virus during this phase was 36.5 h. Using data from days 30, 60, and 90 days as segment 2, the time required for the logarithm of the titers to halve (5.86 log-unit) was calculated to be 52.8 days, with a viral half-life of 144.5 h during this phase.

## 4. Discussion

In this study, we report the detection and characterization of a novel birnavirus strain in mandarin fish, named MFBV. Furthermore, we validated through Koch’s postulates that MFBV is a novel causative agent of disease for mandarin fish. The full-length sequences of MFBV segments A and B were obtained through RACE and molecular cloning.

(1) Genomic features of MFBV

The open reading frames (ORFs) within the MFBV genome were delineated by aligning with reference sequences of IPNV [13], IBDV [14], and BSNV [15], which have been experimentally validated. Overall, the genetic organization of MFBV is similar to that of other birnaviruses, albeit with several distinct exceptions. Firstly, MFBV was distinguished by the presence of the X gene, a feature shared only with a subset of birnaviruses, including LCBV, BSHV, and TV-1 (Figure 4). The specific function of the X gene remains unknown [15], but its presence contributed to a longer length of MFBV segment A compared to other birnaviruses. Secondly, MFBV lacked an overlapping gene named small ORF or VP5, which is approximately 400 bp in length and is present in most birnaviruses, with the exception of LBBV and RBV [16]. Notably, the VP5 corresponding to the small ORF has been confirmed to be expressed in IPNV and IBDV [17,18], but its sequences are non-homologous and its genomic location is irregular. For example, the small ORF in DXV is located between VP4 and VP3 [19], whereas in DBV it was located within VP2 [20] (Figure 4). Thirdly, three small peptides are predicted to be cleaved from MFBV preVP2 during the maturation process, with lengths of 43 aa, 7 aa, and 18 aa. This differs from the four peptides found in BSNV and IBDV (Figure 4). These small peptides are recognized as capsid-associated peptides and have biological roles in deforming and perforating biological membranes [21,22]. The biological roles of the unique X gene, absence of VP5, and variation in preVP2 cleavage peptides in MFBV remain unknown. Whether these proteins are linked to host adaptation, replication efficiency, or immune evasion strategies, and viral survival or virulence requires further investigation.

(2) Phylogenetic relationships and taxonomy

We performed phylogenetic analyses between MFBV and other RNA viruses. MFBV, LBBV, and LCBV formed a distinct branch with the closest phylogenetic relationship, while other birnaviruses classified by ICTV and newly discovered strains are more distantly related (based on RdRp amino acid sequence analysis: Rocky Mountain birnavirus, 47.94% identity; Wenling jack mackerels birnavirus, 48.05% identity; and Wenling Japanese topeshark birnavirus, 50.12% identity).

(3) Conservation of viral enzymatic mechanisms.

MFBV VP4 protease and its polyprotein substrate, exhibit low homology with the other members of the *Birnaviridae* family, which raises questions regarding whether they follow the same cleavage mechanism. To address this, we analyzed the amino acid sequences of MFBV and other birnaviruses VP4 proteins. Alignment of VP4 proteins revealed that the two coordinated amino acids, serine and lysine, are conserved in *Birnaviridae* members. The Ser-Lys catalytic dyad active site in IPNV, IBDV, and BSNV VP4 has been experimentally identified and is considered a serine protease [15,18,23,24], suggesting that MFBV VP4 utilizes a Ser(701)-Lys(738) catalytic dyad (Figure S1A).

Furthermore, we examined the P7-P7′ amino acid residues flanking the cleavage sites of MFBV polyprotein. The analysis was based on the cleavage sites of TV-1 polyprotein, which have been experimentally validated [22]. The results revealed that the conserved motif at the cleavage site is an alanine (Ala) at position P1 (with the exception of the serine (Ser) in DXV), and no other conserved motifs were found. Additionally, the P1′-P3′ sites of the small peptide cleavage sites exhibit conserved ASG amino acid residues, although the function of which remains unknown (Figure S1B). These analyses suggest that although there is significant divergence in birnavirus sequences, the MFBV protease cleavage mechanism is conserved.

RdRp self-guanylylation has been demonstrated in multiple birnaviruses, such as DXV [25], IBDV [26], and IPNV [27]. Specifically, the 5′ ends of the dsRNA genome of birnaviruses are bound to a genome-linked protein (VPg, another form of RdRp) by a Ser-5′-GMP phosphodiester bond at the self-guanylylation site [28]. The guanylylation site of IPNV has been determined to be S163 using peptide digestion and site-directed mutagenesis [29]. Additionally, the IBDV guanylylation site residue was putatively identified as S166 [26], and there have been no other analyses of birnaviruses self-guanylylation sites. We used the IPNV guanylylation site amino acid sequence as a reference to predict the guanylylation site in other birnaviruses through homology comparison. Ser residues were found at the corresponding position or at position -2 in all aligned sequences. These residues are also approximately 80 amino acids away from the conserved motif G, suggesting a topological and functional conservation. Based on these findings, we predict S164 as the site for self-guanylylation in MFBV RdRp (Figure S2).

Another feature of the MFBV or other birnaviruses was that RdRp has unique catalytic core motifs. The prototypic RNA virus RdRp domain harbors seven motifs, which are arranged in the order G, F, A, B, C, D, and E from amino- to carboxy-terminus. The only exception to this scheme can be found in some ssRNA(+) non-segmented viruses and birnavirus [26,30,31], which is exactly the case with MFBV, which was G-F-C-A-B-D-E. (Figure S3A).

Motifs C and A are the most essential catalytic core motifs in RdRp, housed in the palm subdomain of a right-hand architecture [31,32,33]. Within motif C, the DD amino acid residues are in the RNA polymerase active site, allowing catalysis to occur via a two-metal mechanism [31,34]. The DD amino acid motif is conserved across most RNA viruses, including positive-strand RNA viruses (+RNA), segmented negative-strand RNA viruses (seg −RNA), double-strand RNA viruses of the reovirus family (Reo dsRNA), and reverse transcriptases (RT). However, the DD amino acid motif is not found in MFBV; instead, it is replaced by the DN motif (Figure S3A). Furthermore, we conducted multiple sequence alignment analyses on representative RdRp sequences from all eleven families within the order *Mononegavirales* (negative-sense genome single-stranded RNA viruses, including *Artoviridae*, *Bornaviridae*, *Filoviridae*, *Lispiviridae. Mymonaviridae*, *Nyamiviridae*, *Paramyxoviridae*, *Pneumoviridae*, *Rhabdoviridae*, *Sunviridae*, and *Xinmoviridae*; ICTV Virus Taxonomy 2023 Release) to examine the conservation of motif C catalytic core. The results indicate that MFBV employs a DN catalytic core consistent with other birnaviruses and *Mononegavirales* viruses (Figure S3A,B) [25,31]; for instance, in Ebola virus [35], respiratory syncytial virus [36], and rabies virus [37]. The DD amino acid residues in RddRp motif C are involved in coordinating interactions with divalent metal ions, which are essential for the phosphoryl transfer reaction [34]. Some suggest that the distinct evolution of the catalytic core in Birnavirus and *Mononegavirales*, from DD to DN, may have enhanced RdRp adaptability to divalent metal ions. The IBDV DN motif has been shown to exhibit greater activity for nucleotide polymerization when utilizing Mn^2+^, while replacing it with DD leads to more efficient coordination with Mg^2+^ [26,38]. However, the DN motif remains a minority in RNA viruses [31]. Whether this feature confers a survival advantage to birnaviruses such as MFBV in natural environments requires further investigation.

Another highly conserved RdRp motif is the DX2-4D amino acid residues in motif A [31,32]. However, in MFBV, this catalytic motif is replaced by DX2K, a pattern unique to segmented negative-strand RNA viruses (seg −RNA) and non-segmented negative-strand RNA(−RNA) viruses [31]. Furthermore, we found that the DX2K motif of MFBV is completely consistent with that of most birnaviruses and the family *Filoviridae* of order *Mononegavirales*, specifically the DLEK motif. This family includes well-known viruses such as Ebola virus and Marburg virus, as well as fish filoviruses discovered through virus metagenomic studies (Wenling frogfish filovirus strain, MG599980.1; Wenling thamnaconus septentrionalis filovirus, MG599981.1). Both the DN and DLEK catalytic motifs are rare among RNA viruses, the concurrent utilization of these two motifs by both birnaviruses and filoviruses may suggest a potential evolutionary relationship between the two (Figure S3A,B). However, based on capsid topology analysis, the jelly roll structure of IBDV VP2 (within segment A) and its inserted domain were, respectively, linked to two different categories of RNA viruses: black beetle virus (Nodaviridae, non-enveloped positive-strand RNA viruses, T = 3) and rotavirus (Reoviridae, non-enveloped double-stranded RNA viruses, external and intermediate layer, T = 13) [12]. Along with our discovery of the phylogenetic relationship between segment B and filoviruses in this study, this supports the hypothesis that birnavirus segments A and B have undergone reassortment and followed distinct evolutionary pathways [16,39].

The untranslated regions (UTRs) of the birnavirus genome are essential for replication and translation processes [38,40]. The 5′UTR serves as a binding site for VPg [41,42] and the 3′UTR cytosines allow protein-primed initiation of second-strand RNA synthesis [27,38]. The precise sequences of 5′UTR and 3′UTR have been experimentally determined for some birnaviruses, such as LBBV [6], BSNV [15], IPNV [13], IBDV [14], and TV-1 [22]. Multiple sequence alignment analysis of MFBV and these birnaviruses revealed a conserved starting motif of GGAAA (except for IBDV’s GGAUA) (Figure S4). Additionally, the two constitutive cytosines at 3′ terminus (except for LBBV and TV-1 segment B) form a small stem-loop secondary structure, which is crucial for virus replication or virulence [43]. However, the position and length of the 3′UTR stem-loop end are not fixed in birnaviruses (Figure S4).

Birnavirus mRNA is known to lack a cap, thus relying on cap-independent mechanisms for translation initiation. However, the relatively short 5′UTR of birnaviruses appears insufficient to form a typical internal ribosome entry site (IRES) for translation initiation (a cap-independent mechanism) [24]. While there are reports of IPNV segment A having IRES within the 120 bp 5′UTR [5], whether this critical IRES secondary structure is conserved in MFBV remains unknown. We used MXfold2 (https://ws.sato-lab.org/mxfold2/, accessed on 20 May 2025) [44] to predict the MFBV 5′UTR secondary structure. The result revealed the stem-loop structures at the 5′ ends of segments A and B of MFBV, albeit simpler compared to known IRES structures of four classes [45]. Furthermore, MFBV’s segment A 5′UTR is only 170 bp, and segment B 5′UTR is only 96 bp, significantly shorter than compact typical Class III (332 bp) [15] or Class IV intergenic region IRES (191 bp, KP974706.1) structures [28]. Therefore, it is speculated that IRES may not be universally present in birnaviruses, at least not in MFBV, suggesting that MFBV may initiate translation through alternative pathways (Figure S5).

(4) Biological Characteristics of MFBV

MFBV exhibits a replication cycle of 8–10 h at 27 °C in SCK cells. For IPNV, the complete replication cycle takes about 24 h at 15 °C, but is shortened to 16–20 h at 22 °C in salmon embryo (CHSE-214) cells, with replication ceasing at 28 °C [46,47,48]. At their respective suitable replication temperatures, MFBV replicates significantly faster than IPNV. Furthermore, approximately 7.94 × 10^7^ MFBV RNA copies per μg total RNA can be detected in the spleen as early as 16 hpi, which means MFBV replication is also notably faster than other pathogens of mandarin fish, such as ISKNV [49] and MRV [9]. The rapid replication kinetics may explain why severe symptoms, including mortality, occur as early as 6 h post-infection in mandarin fish. In contrast, other mandarin fish viral pathogens such as ISKNV and MRV typically require 3–5 days to induce mortality even at high inoculum doses [9,49]. In the artificial infection experiments of LBBV in bass, a similar rapid mortality was observed, with deaths occurring after 24 hpi, reaching 100% by 3 dpi. This reflects the rapid replication dynamics of birnavirus and its high virulence to juvenile fish in acute infection. However, MFBV infection in mandarin fish caused mortality as early as 6 hpi, which could be attributed to the higher infectious dose used in this study, as well as potential differences between viral strains and host species. Further, in all fourteen tissues, the MFBV RNA copies per μg total RNA exceeded 7.94 × 10^6^, with only a tenfold difference between the tissue with the highest (spleen) and the lowest (gonad). This suggests that MFBV is capable of causing systemic infections that involve multiple organs. It is speculated that severe systemic infection is another factor contributing to the rapid mortality. There is evidence that certain birnaviruses, such as IPNV, LMBV, and MABV can cause high mortality rates in artificial infections. For example, IPNV was shown to cause mortality rates of approximately 80% in experimental infections [50]. Similarly, studies on LBBV have also documented its high virulence on largemouth bass [6] and marine birnavirus (MABV) on fingerlings of rock bream [51].

Due to the rapid onset of disease and death, inflammatory responses such as immune cell infiltration were not identified in the sections, although cell death was clearly observed. Overall, MFBV caused severe damage to the spleen, kidney, and intestines. Additionally, in the fry immersion infection experiment, the 19.9% mortality observed in the fry control group is consistent with natural patterns observed in fish breeding. In field breeding of mandarin fish, similar losses are often observed due to factors such as handling stress, cannibalism, developmental abnormalities, and other environmental stressors. This is also the difficulty in mandarin fish farming. To enhance the robustness of our results, the immersion challenge experiment was conducted in three independent trials. The important thing is that, statistical analysis of survival data, as provided in our initial response, confirmed significant differences between the treatment and control groups in all three trials. It is noteworthy that the threat of MFBV to fry is greater than that to juveniles, as evidenced by the higher mortality rate of fry (approximately 30–40% higher).

To mitigate MFBV outbreaks, we tested its sensitivity to various disinfectants. The experimental concentration ranges of disinfectants in this study were determined based on the study on ISKNV and *Micropterus salmoides* rhabdovirus. ISKNV can be inactivated by sodium hypochlorite (1000 ppm for 30 min) and benzalkonium chloride (650 ppm for 10 min) [52], while *Micropterus salmoides* rhabdovirus can be inactivated by 500 ppm povidone-iodine and 500 ppm glutaraldehyde within 30 min [53]. Based on these results presented, one or more disinfectants can be chosen for different epidemic outbreaks. Additionally, in the laboratory, 15-day storage reduced MFBV infectivity by 99.9%, suggesting natural decay could be an alternative in the absence of disinfection. However, organic and inorganic substances in natural water may affect decay rates [54], requiring further optimization.

Taken together, this research may have important implications for exploring the MFBV replication mechanism and developing disease containment strategies.

## Data Availability

The cell line metadata presented in this study are openly available in the NCBI BioSample database. SCK cell line: [accession number: SAMN38845202] (link: https://www.ncbi.nlm.nih.gov/biosample/38845202) (14 December 2023). The MFBV genome accession numbers are PP786692.1 (https://www.ncbi.nlm.nih.gov/nuccore/2733894183) (22 May 2024) and PP786693.1 (https://www.ncbi.nlm.nih.gov/nuccore/2733894185) (22 May 2024).

## Supplementary Materials

The following supporting information can be downloaded at: https://www.mdpi.com/article/10.3390/genes16060629/s1, Figure S1. (A) The catalytic dyad of VP4. The amino acid sequence of VP4 was aligned using Kalign. IPNV, IBDV, and BSNV VP4 served as reference for homology comparison to predict the catalytic site of MFBV VP4. The catalytic dyad of VP4 is highlighted in orange. (B) The conserved amino acid residues of polyprotein cleavage sites. Scissors indicate the predicted cleavage sites of the MFBV polyprotein. The conserved amino acid is indicated below. Completely conserved sites are highlighted in orange, positions with two types of amino acids are shown in yellow, and positions with three amino acids are highlighted in green. Figure S2. Guanylylation sites of MFBV and other birnaviruses are highlighted in orange, predicted based on homology alignment with the guanylylation site of IPNV RdRp. Figure S3. (A) Key conserved residues of the RdRp motifs. The RdRp amino acid sequence was of aligned using Kalign, the numbers indicating the positions of the motifs. Completely conserved sites are highlighted in orange, positions with two types of amino acids are highlighted in yellow, and positions with three amino acids are shown in green. (B) Comparison of catalytic core amino acids in motifs A and C between birnaviruses and Mononegavirales viruses. The data include representative viruses from all 11 families of the order Mononegavirales and representative viruses from 7 genera of the Filoviridae family. Completely conserved sites between Mononegavirales viruses and birnaviruses are highlighted in orange, residues conserved only within each set are highlighted in yellow. The sequence identifiers are arranged as family, genus, strain and accession number. If there is no genus, then only the strain name is displayed. Figure S4. UTR sequences of birnaviruses. Conserved motifs are highlighted in orange. Figure S5. Secondary structure prediction of birnavirus UTRs. The data includes only virus UTR sequences that have been determined through experimental methods. Table S1: Supplementary Table S1.
