# Supplementary material for: Genomic Characterization and Pathogenicity of a Novel Birnavirus Strain Isolated from Mandarin Fish (Siniperca chuatsi)
Source: Genes (Basel). 2025 May 24;16(6):629. doi: 10.3390/genes16060629 (PMC12191561; doi:10.3390/genes16060629)
Supplement: Supplementary file 1 [file genes-16-00629-s001.zip › genes-3644508-supplementary.pdf]

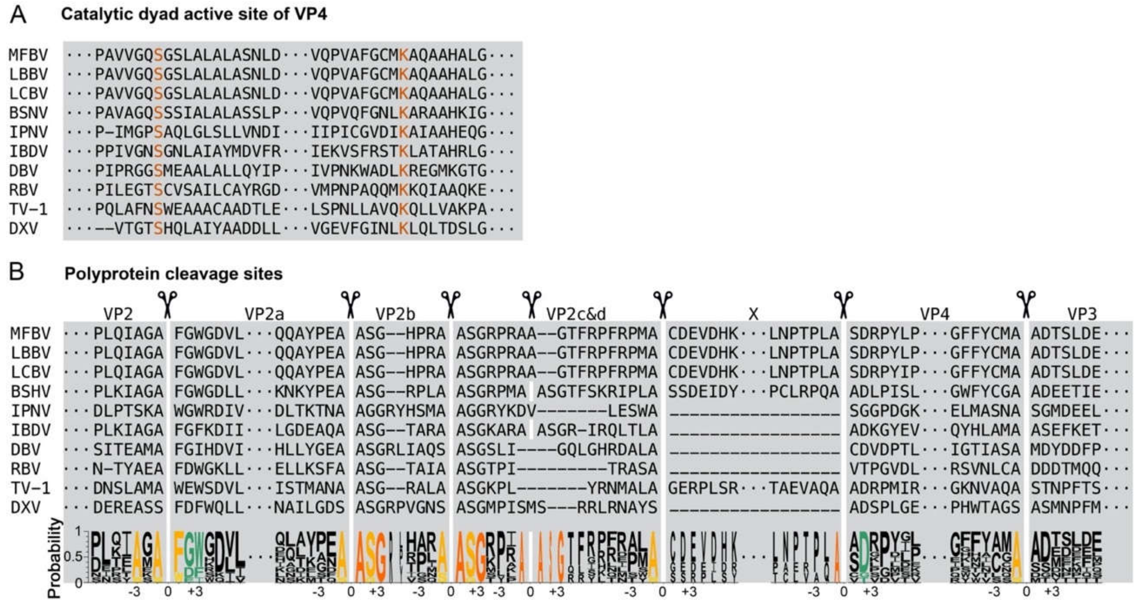

Figure S1. (A) The catalytic dyad of VP4. The amino acid sequence of VP4 was aligned using Kalign. IPNV, IBDV, and BSNV VP4 served as reference for homology comparison to predict the catalytic site of MFBV VP4. The catalytic dyad of VP4 is highlighted in orange. (B) The conserved amino acid residues of polyprotein cleavage sites. Scissors indicate the predicted cleavage sites of the MFBV polyprotein. The conserved amino acid is indicated below. Completely conserved sites are highlighted in orange, positions with two types of amino acids are shown in yellow, and positions with three amino acids are highlighted in green.

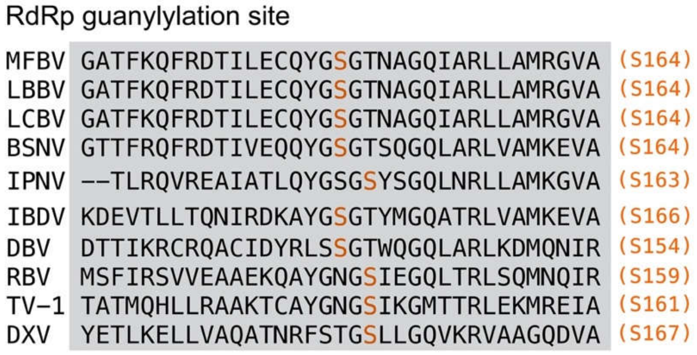

Figure S2. Guanylylation sites of MFBV and other birnaviruses are highlighted in orange, predicted based on homology alignment with the guanylylation site of IPNV RdRp.

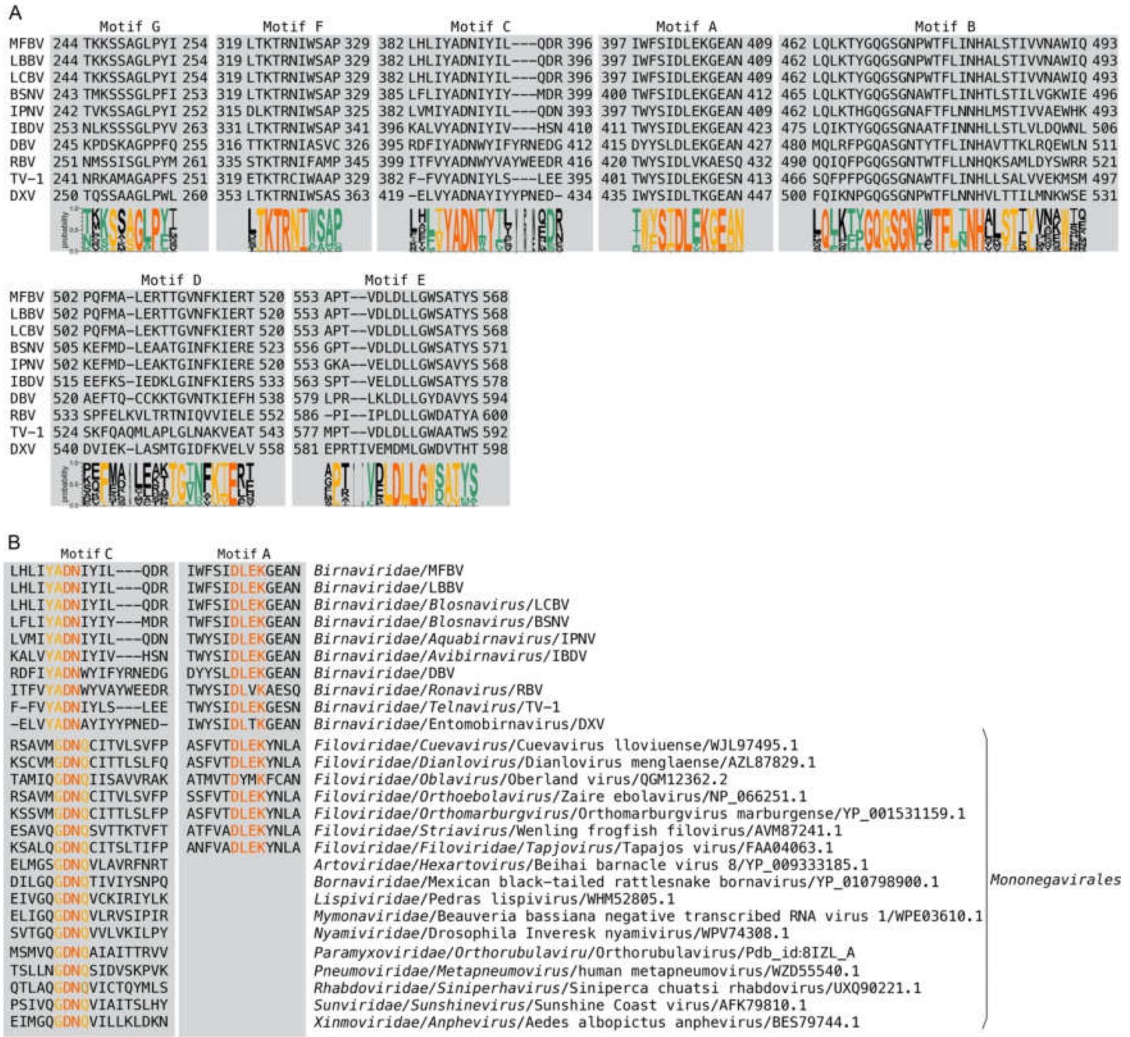

Figure S3. (A)Key conserved residues of the RdRp motifs. The RdRp amino acid sequence was of aligned using Kalign, the numbers indicating the positions of the motifs. Completely conserved sites are highlighted in orange, positions with two types of amino acids are highlighted in yellow, and positions with three amino acids are shown in green. (B) Comparison of catalytic core amino acids in motifs A and C between birnaviruses and Mononegavirales viruses. The data include representative viruses from all 11 families of the order Mononegavirales and representative viruses from 7 genera of the Filoviridae family. Completely conserved sites between Mononegavirales viruses and birnaviruses are highlighted in orange, residues conserved only within each set are highlighted in yellow. The sequence identifiers are arranged as family, genus, strain and accession number. If there is no genus, then only the strain name is displayed.

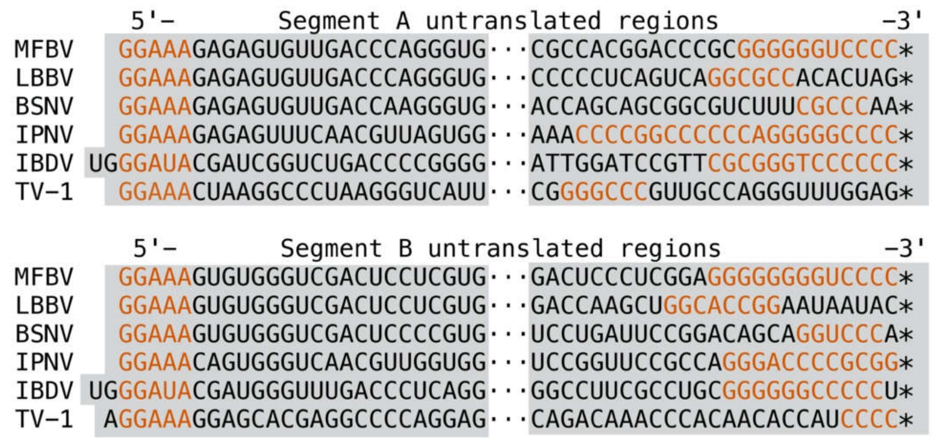

Figure S4. UTR sequences of birnaviruses. Conserved motifs are highlighted in orange.

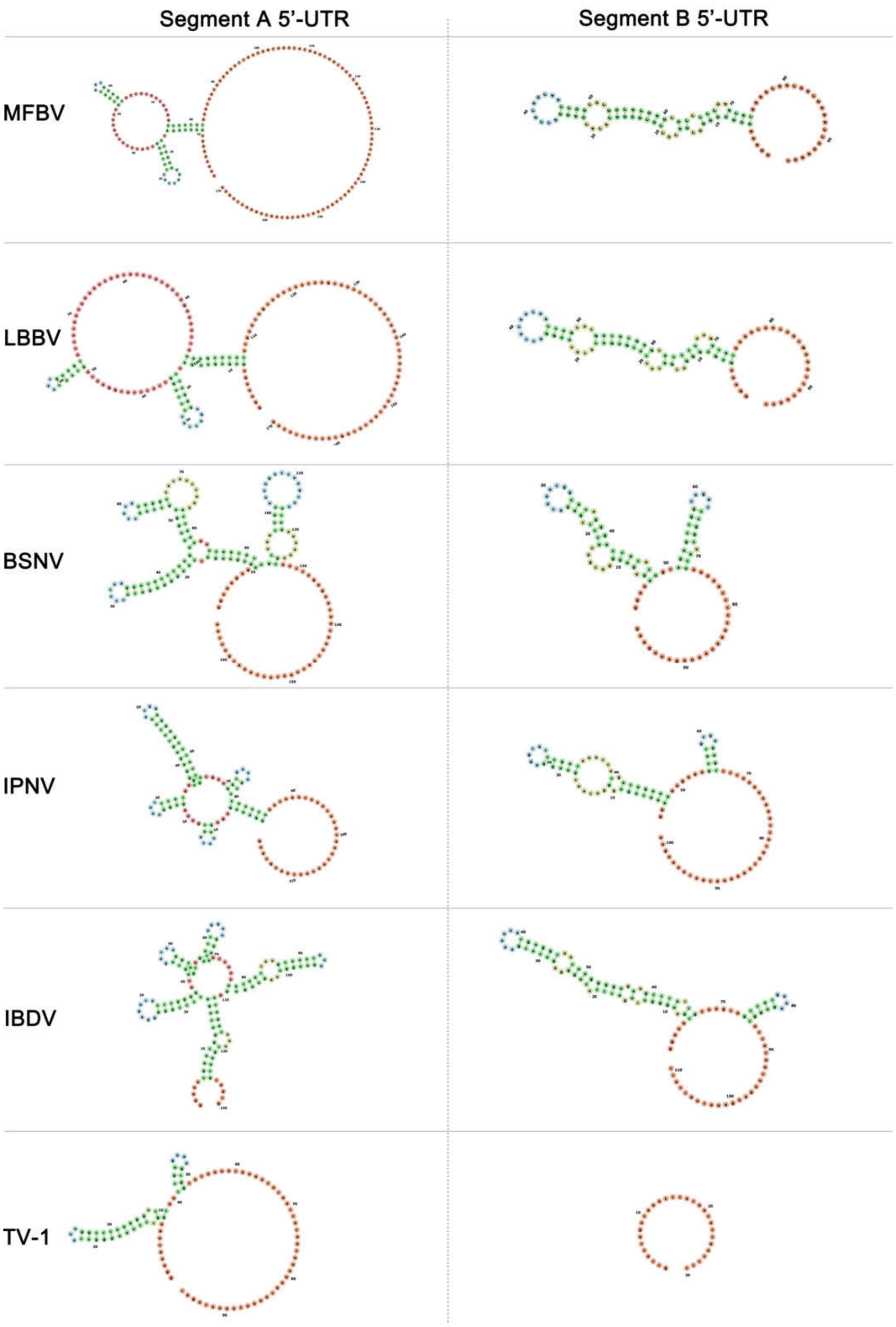

Figure S5. Secondary structure prediction of birnavirus UTRs. The data includes only virus UTR sequences that have been determined through experimental methods.
